# Supplementary material for: A randomized controlled trial evaluating stress arousal reappraisal and worked example effects on psychophysiological responses during breaking bad news
Source: Sci Rep. 2025 Jul 2;15:23290. doi: 10.1038/s41598-025-06995-7 (PMC12222752; doi:10.1038/s41598-025-06995-7)
Supplement: Supplementary file 1 — Supplementary Material 1 [file 41598_2025_6995_MOESM1_ESM.pdf]

A randomized controlled trial evaluating stress arousal reappraisal and worked example effects on psychophysiological responses during breaking bad news

Supplementary Material

Michel Bosshard[1,2*], Sissel Guttormsen[1], Urs Markus Nater[3,4], Felix Schmitz[1 †], Patrick Gomez[5 †], Christoph Berendonk[1 †]

[1]Institute for Medical Education, University of Bern, Bern, Switzerland

[2]Graduate School for Health Sciences, University of Bern, Bern, Switzerland

[3]Department of Clinical and Health Psychology, University of Vienna, Vienna, Austria

[4]University Research Platform “Stress of life (SOLE) – Processes and Mechanisms underlying everyday Life Stress”, University of Vienna, Vienna, Austria

[5] Department of Occupational and Environmental Health, Unisanté, Center for Primary Care and Public Health & University of Lausanne, Lausanne, Switzerland

* Corresponding Author

† Felix Schmitz, Patrick Gomez and Christoph Berendonk share last authorship

Email: michel.bosshard@unibe.ch

Supplementary Table S1

	SAR-only (n = 55)	WE-only (n = 58)	SAR & WE (n = 57)	No-intervention (n = 59)	Effect
BMI (kg/m^2)	22.29 (2.89)	22.12 (2.64)	22.39 (2.69)	22.49 (2.97)	$F(3, 225) = 0.18, p = .91$
Sex (% female)	70.9	69.0	68.4	67.8	X^2 $(3, N = 229) = 0.14, p = .99$
Age (in years)	22.42 (2.17)	22.24 (1.33)	22.60 (1.81)	22.42 (1.94)	$F(3, 225) = 0.36, p = .78$
Shift work (% yes)	27.3	29.3	19.3	27.1	X^2 $(3, N = 229) = 1.75, p = .63$
Contraceptives (% yes)	36.4	36.2	29.8	35.6	X^2 $(3, N = 229) = 0.74, p = .86$
Depression	2.7 (2.9)	1.9 (2.0)	2.7 (3.1)	2.5 (2.6)	$F(3, 225) = 1.25, p = .29$
Anxiety	2.4 (3.0)	2.9 (2.5)	2.6 (2.5)	2.6 (2.8)	$F(3, 225) = 0.31, p = .82$
Stress	4.4 (3.4)	4.2 (3.4)	3.6 (3.6)	4.7 (3.7)	$F(3, 225) = 0.96, p = .41$
BBN skills	4.1 (0.9)	4.1 (1.0)	3.9 (0.9)	3.9 (0.9)	$F(3, 187) = 0.57, p = .64$
BBN experience (% yes)	18.2	6.9	8.8	8.5	X^2 $(3, N = 229) = 4.70, p = .20$
BBN interest	5.9 (0.9)	5.8 (0.9)	5.9 (0.8)	5.6 (0.9)	$F(3, 225) = 1.48, p = .22$
BBN motivation	6.5 (0.6)	6.0 (0.9)	6.3 (0.7)	6.3 (0.9)	$F(3, 225) = 3.89, p = .010$
BBN task duration (in seconds)	528 (113)	506 (105)	507 (110)	494 (121)	$F(3, 225) = 0.89, p = .45$

Sociodemographic and BBN-related variables for the four experimental groups. Mean values with standard deviation in parentheses. BMI = Body Mass Index.

Supplementary Table S2

Variable	Period	SAR-only (n = 55)	WE-only (n = 58)	SAR & WE (n = 57)	No-intervention (n = 59)	Effect
Resources-Demands differential	Baseline	-0.84 (0.17)	-0.97 (0.18)	-1.05 (0.14)	-0.85 (0.20)	$F(3, 225) = 0.51$ $p = .68$
	Post-intervention	-0.85 (0.14)	-1.05 (0.17)	-0.96 (0.13)	-1.07 (0.19)	
	Post-BBN	-1.09 (0.21)	-1.02 (0.19)	-0.84 (0.18)	-1.58 (0.19)	
Demand evaluation	Baseline	4.38 (0.11)	4.41 (0.11)	4.51 (0.08)	4.32 (0.11)	$F(3, 225) = 0.56$ $p = .65$
	Post-intervention	4.56 (0.11)	4.62 (0.11)	4.67 (0.09)	4.51 (0.09)	
	Post-BBN	4.53 (0.16)	4.48 (0.13)	4.46 (0.13)	4.66 (0.11)	
Resource evaluation	Baseline	3.55 (0.12)	3.45 (0.12)	3.40 (0.11)	3.47 (0.13)	$F(3, 225) = 0.23$ $p = .87$
	Post-intervention	3.71 (0.11)	3.57 (0.11)	3.70 (0.10)	3.44 (0.12)	
	Post-BBN	3.44 (0.14)	3.47 (0.11)	3.61 (0.11)	3.08 (0.11)	

Descriptive statistics of the resource and demand outcomes for the four experimental groups. Raw mean values with standard error in parentheses.

Supplementary Table S3

Variable	Period	SAR-only (n = 48)	WE-only (n = 49)	SAR & WE (n = 44)	No-intervention (n = 48)	Effect
Cardiovascular index	Baseline	-0.17 (0.33)	-0.11 (0.28)	0.29 (0.24)	0.01 (0.24)	F(3, 185) = 0.52 p = .67
	Pre-BBN	0.01 (0.30)	-0.15 (0.31)	0.31 (0.19)	-0.28 (0.27)	
	BBN	0.53 (0.29)	-0.37 (0.29)	0.52 (0.22)	-0.62 (0.25)	
	Post-BBN	0.06 (0.32)	0.00 (0.26)	0.35 (0.23)	-0.38 (0.29)	
Cardiac output (in L/min)	Baseline	6.03 (0.20)	6.05 (0.16)	6.25 (0.17)	6.12 (0.16)	F(3, 185) = 0.31, p = .82
	Pre-BBN	6.73 (0.21)	6.80 (0.22)	6.90 (0.16)	6.45 (0.21)	
	BBN	7.51 (0.25)	6.85 (0.19)	7.43 (0.19)	6.44 (0.19)	
	Post-BBN	6.14 (0.22)	6.06 (0.16)	6.21 (0.17)	5.72 (0.18)	
Total peripheral resistance (in mmHg·min/L)	Baseline	15.65 (0.76)	15.45 (0.65)	14.49 (0.46)	15.22 (0.48)	F(3, 185) = 0.67, p = .57
	Pre-BBN	17.34 (0.80)	17.72 (0.82)	16.49 (0.43)	17.74 (0.65)	
	BBN	17.28 (0.62)	19.33 (0.81)	17.07 (0.51)	19.18 (0.61)	
	Post-BBN	19.65 (0.89)	19.62 (0.77)	18.36 (0.60)	20.27 (0.84)	

Supplementary Table S3 (continued)

Variable	Period	SAR-only (n = 48)	WE-only (n = 49)	SAR & WE (n = 44)	No-intervention (n = 48)	Effect
Stroke Volume (in mL)	Baseline	80.81 (2.81)	78.14 (2.12)	77.77 (1.93)	78.38 (2.28)	F(3, 185) = 0.35, p = .79
	Pre-BBN	75.90 (2.53)	82.73 (1.98)	73.02 (1.92)	70.96 (2.45)	
	BBN	78.25 (2.74)	71.61 (2.07)	75.45 (2.00)	68.48 (2.23)	
	Post-BBN	83.21 (3.26)	79.52 (2.50)	77.43 (2.35)	76.70 (2.98)	
Heart Rate (in bpm)	Baseline	75.85 (1.93)	78.41 (1.85)	80.98 (1.87)	79.29 (1.88)	F(3, 185) = 1.26, p = .29
	Pre-BBN	89.98 (2.31)	94.71 (2.92)	96.02 (2.45)	92.60 (2.45)	
	BBN	97.67 (2.59)	96.73 (1.94)	99.73 (2.31)	96.02 (2.59)	
	Post-BBN	75.31 (2.13)	77.14 (1.65)	81.57 (2.04)	77.29 (2.25)	
Pre-Ejection Period (in ms)	Baseline	117.60 (2.12)	114.67 (2.37)	112.52 (2.12)	107.81 (2.68)	F(3, 185) = 3.15, p = .026
	Pre-BBN	98.67 (3.25)	96.57 (3.02)	90.16 (3.52)	94.73 (3.36)	
	BBN	85.44 (3.09)	88.31 (3.07)	84.11 (3.10)	87.63 (3.10)	
	Post-BBN	101.75 (2.62)	105.37 (2.36)	101.30 (2.29)	99.77 (3.13)	

Descriptive statistics of the cardiovascular outcomes for the four experimental groups. Raw mean values with standard error in parentheses.

Supplementary Table S4

Fixed Effect	Model 1			Model 2		
	Beta	*SE*	*p*	Beta	*SE*	*p*
Intercept	3.96	0.45	< .001	3.96	0.45	< .001
SAR [a]	0.05	0.14	.72	0.05	0.15	.76
WE [b]	0.11	0.14	.43	0.11	0.15	.48
Post-Intervention [c]	0.76	0.41	.062	0.75	0.41	.069
Post-BBN [c]	0.67	0.55	.23	0.69	0.56	.22
Motivation [d]	0.06	0.07	.42	0.06	0.07	.42
SAR × WE	0.02	0.18	.89	0.03	0.21	.89
SAR × Post-Intervention	-0.00	0.10	.97	0.02	0.14	.91
SAR× Post-BBN	-0.14	0.13	.29	-0.18	0.19	.34
WE × Post-Intervention	-0.02	0.10	.82	-0.00	0.14	.98
WE × Post-BBN	-0.25	0.13	.064	-0.29	0.19	.13
Motivation × Post-Intervention	-0.09	0.06	.16	-0.09	0.06	.16
Motivation × Post-BBN	-0.05	0.09	.52	-0.06	0.09	.52
SAR × WE × Post-Intervention				-0.04	0.19	.85
SAR × WE × Post-BBN				0.08	0.26	.77

Linear mixed effects regressions for the demand evaluation. [a] No-SAR = 0, SAR = 1. [b] No-WE = 0, WE = 1. [c] Reference = baseline. [d] Motivation is grand mean centered.

Supplementary Table S5

Fixed Effect	Model 1			Model 2		
	Beta	*SE*	*p*	Beta	*SE*	*p*
Intercept	2.53	0.51	< .001	2.54	0.52	< .001
SAR [a]	0.06	0.15	.72	0.04	0.17	.83
WE [b]	0.03	0.15	.84	0.01	0.17	.94
Post-Intervention [c]	-0.40	0.52	.45	-0.41	0.53	.44
Post-BBN [c]	-0.51	0.59	.38	-0.54	0.59	.37
Motivation [d]	0.15	0.08	.062	0.15	0.08	.063
SAR × WE	-0.16	0.18	.36	-0.13	0.24	.60
SAR × Post-Intervention	0.17	0.13	.17	0.18	0.18	.30
SAR × Post-BBN	0.23	0.14	.10	0.28	0.20	.17
WE × Post-Intervention	0.16	0.13	.21	0.17	0.18	.33
WE × Post-BBN	**0.37**	**0.14**	**.009**	0.41	0.20	.037
Motivation × Post-Intervention	0.06	0.08	.47	0.06	0.08	.47
Motivation × Post-BBN	0.02	0.09	.80	0.02	0.09	.80
SAR × WE × Post-Intervention				-0.02	0.25	.92
SAR × WE × Post-BBN				-0.09	0.28	.75

Linear mixed effects regressions for the resource evaluation. Significant interaction effects ($p < .05$) are presented in bold. [a] No-SAR = 0, SAR = 1. [b] No-WE = 0, WE = 1. [c] Reference = baseline. [d] Motivation is grand mean centered.

Supplementary Table S6

Fixed Effect	Model 1			Model 2		
	Beta	*SE*	*p*	Beta	*SE*	*p*
Intercept	6.04	0.18	< .001	6.12	0.18	< .001
SAR [a]	0.07	0.25	.77	-0.10	0.26	.70
WE [b]	0.11	0.25	.66	-0.06	0.25	.82
Pre-BBN [c]	0.44	0.11	< .001	0.33	0.13	.011
BBN [c]	0.51	0.12	< .001	0.32	0.13	.016
Post-BBN [c]	-0.26	0.10	.007	-0.38	0.11	< .001
Motivation [d]	0.04	0.12	.76	0.04	0.12	.76
SAR × WE	-0.06	0.34	.86	0.28	0.36	.43
SAR × Pre-BBN	0.14	0.13	.29	0.37	0.18	.043
SAR × BBN	**0.77**	**0.14**	**< .001**	1.15	0.19	< .001
SAR × Post-BBN	**0.26**	**0.11**	**.025**	0.49	0.16	.002
WE × Pre-BBN	0.19	0.13	.15	0.42	0.18	.022
WE × BBN	0.11	0.14	.43	0.49	0.19	.009
WE × Post-BBN	0.09	0.11	.42	0.32	0.16	.039
Motivation × Pre-BBN	-0.02	0.08	.77	-0.02	0.08	.77
Motivation × BBN	0.02	0.09	.82	0.02	0.08	.82
Motivation × Post-BBN	-0.03	0.07	.70	-0.03	0.07	.71
SAR × WE × Pre-BBN				-0.47	0.26	.070
SAR × WE × BBN				**-0.78**	**0.26**	**.003**
SAR × WE × Post-BBN				**-0.47**	**0.22**	**.032**

Linear mixed effects regressions for cardiac output. Significant interaction effects ($p < .05$) are presented in bold. [a] No-SAR = 0, SAR = 1. [b] No-WE = 0, WE = 1. [c] Reference = baseline. [d] Motivation is grand mean centered.

Supplementary Table S7

Contrast	Beta	*SE*	*t*(550)	*p*
SAR × WE × BBN				
SAR-only vs. No-intervention (SAR x BBN effect when WE = 0)	**1.15**	**0.19**	**6.17**	**< .001**
SAR & WE vs. WE-only (SAR x BBN effect when WE = 1)	0.37	0.19	1.95	.051
WE-only vs. No-intervention (WE x BBN effect when SAR = 0)	**0.49**	**0.19**	**2.63**	**.009**
SAR & WE vs. SAR-only (WE x BBN effect when SAR = 1)	-0.29	0.19	-1.54	.12
SAR × WE × post-BBN				
SAR-only vs. No-intervention (SAR x post-BBN effect when WE = 0)	**0.49**	**0.16**	**3.12**	**.002**
SAR & WE vs. WE-only (SAR x post-BBN effect when WE = 1)	0.02	0.16	0.10	.92
WE-only vs. No-intervention (WE x post-BBN effect when SAR = 0)	**0.32**	**0.16**	**2.07**	**.039**
SAR & WE vs. SAR-only (WE x post-BBN effect when SAR = 1)	-0.15	0.16	-0.95	.34

Post-hoc analysis of the significant SAR × WE × BBN and SAR × WE × post-BBN interaction for cardiac output. The estimates represent the differences in the changes from baseline to BBN task and from baseline to post-BBN of the two groups of each contrast. Significant effects ($p < .05$) are presented in bold.

Supplementary Table S8

Fixed Effect	Model 1			Model 2		
	Beta	*SE*	*p*	Beta	*SE*	*p*
Intercept	15.36	0.67	< .001	15.23	0.68	< .001
SAR [a]	0.03	0.94	.97	0.31	0.98	.75
WE [b]	0.08	0.93	.93	0.35	0.97	.72
Pre-BBN [c]	2.38	0.36	< .001	2.52	0.42	< .001
BBN [c]	3.70	0.35	< .001	3.96	0.41	< .001
Post-BBN [c]	4.95	0.45	< .001	5.01	0.52	< .001
Motivation [d]	0.40	0.44	.37	0.40	0.44	.37
SAR × WE	-0.84	1.27	.51	-1.40	1.38	.31
SAR × Pre-BBN	-0.57	0.43	.18	-0.85	0.60	.15
SAR × BBN	**-1.76**	**0.42**	**< .001**	-2.27	0.58	< .001
SAR × Post-BBN	-0.88	0.53	.099	-1.02	0.74	.17
WE × Pre-BBN	0.04	0.43	.92	-0.24	0.59	.69
WE × BBN	0.36	0.42	.38	-0.14	0.58	.81
WE × Post-BBN	-0.28	0.53	.60	-0.41	0.73	.57
Motivation × Pre-BBN	0.06	0.27	.84	0.06	0.27	.84
Motivation × BBN	-0.20	0.26	.44	-0.20	0.26	.44
Motivation × Post-BBN	-0.02	0.33	.95	-0.02	0.33	.95
SAR × WE × Pre-BBN				0.57	0.84	.50
SAR × WE × BBN				1.04	0.82	.21
SAR × WE × Post-BBN				0.28	1.04	.79

Linear mixed effects regressions for total peripheral resistance. Significant interaction effects ($p < .05$) are presented in bold. [a] No-SAR = 0, SAR = 1. [b] No-WE = 0, WE = 1. [c] Reference = baseline. [d] Motivation is grand mean centered.

Supplementary Table S9

Fixed Effect	Model 1			Model 2		
	Beta	*SE*	*p*	Beta	*SE*	*p*
Intercept	77.83	2.32	< .001	78.37	2.36	< .001
SAR [a]	3.61	3.26	.27	2.54	3.37	.45
WE [b]	0.74	3.24	.82	-0.33	3.35	.92
Pre-BBN [c]	-6.97	1.10	< .001	-7.42	1.27	< .001
BBN [c]	-9.13	1.19	< .001	-9.90	1.37	< .001
Post-BBN [c]	-0.36	1.46	.80	-1.65	1.68	.33
Motivation [d]	-0.32	1.52	.83	-0.33	1.52	.83
SAR × WE	-4.99	4.42	.26	-2.81	4.77	.56
SAR × Pre-BBN	1.80	1.30	.17	2.70	1.81	.14
SAR × BBN	**5.79**	**1.41**	**< .001**	7.32	1.95	< .001
SAR × Post-BBN	1.53	1.73	.37	4.09	2.39	.088
WE × Pre-BBN	0.93	1.30	.48	1.82	1.80	.31
WE × BBN	1.86	1.40	.19	3.38	1.94	.082
WE × Post-BBN	-0.12	1.72	.94	2.42	2.38	.31
Motivation × Pre-BBN	-0.64	0.82	.44	-0.64	0.82	.44
Motivation × BBN	0.03	0.88	.97	0.03	0.88	.97
Motivation × Post-BBN	-0.14	1.08	.90	-0.14	1.08	.89
SAR × WE × Pre-BBN				-1.84	2.56	.47
SAR × WE × BBN				-3.12	2.76	.26
SAR × WE × Post-BBN				-5.20	3.37	.12

Linear mixed effects regressions for stroke volume. Significant interaction effects ($p < .05$) are presented in bold. [a] No-SAR = 0, SAR = 1. [b] No-WE = 0, WE = 1. [c] Reference = baseline. [d] Motivation is grand mean centered.

Supplementary Table S10

Fixed Effect	Model 0			Model 1			Model 2		
	Beta	*SE*	*p*	Beta	*SE*	*p*	Beta	*SE*	*p*
Intercept	76.99	1.71	< .001	79.10	2.03	< .001	79.30	2.08	< .001
SAR [a]	0.53	1.97	.79	-3.23	2.85	.26	-3.62	2.97	.22
WE [b]	2.72	1.96	.17	-0.33	2.83	.91	-0.71	2.95	.81
Pre-BBN [c]	**14.70**	**0.93**	**< .001**	13.83	1.61	< .001	13.32	1.86	< .001
BBN [c]	**18.90**	**0.79**	**< .001**	17.88	1.36	< .001	16.73	1.57	< .001
Post-BBN [c]	-0.84	0.61	.17	-2.10	1.06	.048	-2.00	1.22	.10
Motivation [d]	0.58	1.23	.64	0.58	1.34	.66	0.58	1.34	.67
SAR × WE				5.21	3.85	.18	6.00	4.19	.15
SAR × Pre-BBN				-0.32	1.91	.87	0.70	2.66	.79
SAR × BBN				2.65	1.62	.10	4.94	2.24	.028
SAR × Post-BBN				1.73	1.25	.17	1.54	1.74	.38
WE × Pre-BBN				2.09	1.90	.27	3.10	2.64	.24
WE × BBN				-0.54	1.61	.74	1.73	2.22	.44
WE × Post-BBN				0.86	1.25	.49	0.66	1.73	.70
Motivation × Pre-BBN				0.36	1.20	.77	0.36	1.20	.77
Motivation × BBN				0.45	1.01	.66	0.45	1.01	.65
Motivation × Post-BBN				-0.25	0.79	.75	-0.25	0.79	.75
SAR × WE × Pre-BBN							-2.07	3.76	.58
SAR × WE × BBN							-4.66	3.16	.14
SAR × WE × Post-BBN							0.40	2.46	.87

Linear mixed effects regressions for heart rate**.** Significant main effects in Model 0 ($p < .05$) are presented in bold. [a] No-SAR = 0, SAR = 1. [b] No-WE = 0, WE = 1. [c] Reference = baseline. [d] Motivation is grand mean centered.

Supplementary Table S11

Fixed Effect	Model 0			Model 1			Model 2		
	Beta	*SE*	*p*	Beta	*SE*	*p*	Beta	*SE*	*p*
Intercept	112.95	2.13	< .001	108.89	2.58	< .001	107.82	2.67	< .001
SAR [a]	-0.54	2.37	.82	7.47	3.57	.038	9.61	3.81	.013
WE [b]	0.99	2.37	.67	4.91	3.55	.17	7.03	3.79	.065
Pre-BBN [c]	**-18.03**	**1.39**	**< .001**	-13.47	2.38	< .001	-13.08	2.74	< .001
BBN [c]	**-26.75**	**1.27**	**< .001**	-22.60	2.17	< .001	-20.16	2.49	**< .001**
Post-BBN [c]	**-11.10**	**1.15**	**< .001**	-9.48	1.95	< .001	-8.04	2.24	**< .001**
Motivation [d]	1.36	1.49	.36	0.59	1.72	.73	0.59	1.72	.73
SAR × WE				-7.59	4.62	.10	-11.94	5.38	.028
SAR × Pre-BBN				-5.23	2.82	.064	-6.01	3.91	.13
SAR × BBN				**-7.88**	**2.57**	**.002**	-12.75	3.55	< .001
SAR × Post-BBN				**-5.05**	**2.31**	**.029**	-7.95	3.21	.014
WE × Pre-BBN				-4.10	2.81	.15	-4.87	3.89	.21
WE × BBN				-0.63	2.56	.81	-5.46	3.53	.12
WE × Post-BBN				1.72	2.30	.45	-1.14	3.19	.72
Motivation × Pre-BBN				0.49	1.77	.78	0.49	1.77	.78
Motivation × BBN				2.48	1.61	.12	2.48	1.60	.12
Motivation × Post-BBN				0.43	1.45	.77	0.43	1.45	.77
SAR × WE × Pre-BBN							1.59	5.52	.77
SAR × WE × BBN							**9.93**	**5.02**	**.048**
SAR × WE × Post-BBN							5.89	4.53	.19

Linear mixed effects regressions for pre-ejection period. Significant main effects in Model 0 and interaction effects in Models 1 and 2 ($p < .05$) are presented in bold. [a] No-SAR = 0, SAR = 1. [b] No-WE = 0, WE = 1. [c] Reference = baseline. [d] Motivation is grand mean centered.

Supplementary Table S12

Contrast	Beta	*SE*	*t*(552)	*p*
SAR-only vs. No-intervention (SAR x BBN effect when WE = 0)	**-12.75**	**3.55**	**-3.59**	**< .001**
SAR & WE vs. WE-only (SAR x BBN effect when WE = 1)	-2.83	3.61	-0.78	.43
WE-only vs. No-intervention (WE x BBN effect when SAR = 0)	-5.46	3.53	-1.55	.12
SAR & WE vs. SAR-only (WE x BBN effect when SAR = 1)	4.47	3.62	1.23	.22

Post-hoc analysis of the significant SAR × WE × BBN interaction for the pre-ejection period. The estimates represent the differences in the changes from baseline to BBN task of the two groups of each contrast. Significant effects ($p < .05$) are presented in bold.

Supplementary Figure 1

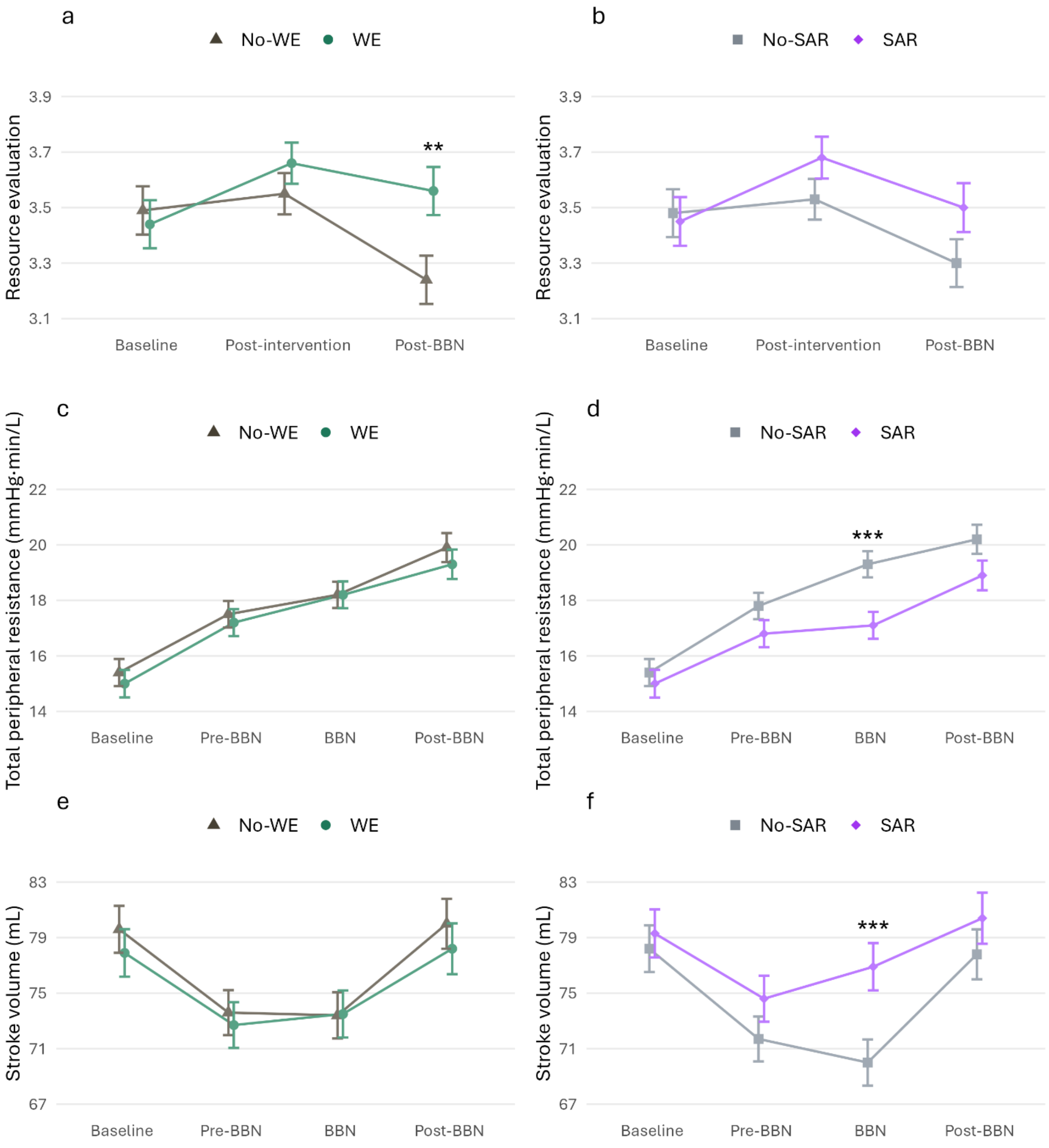

Line plots of Model 1 estimated marginal means of resource evaluation, total peripheral resistance, and stroke volume for WE vs. No-WE groups (a, c, e) and SAR vs. No-SAR groups (b, d, f). The error bar represents the standard error. Significant differences in the changes from baseline to the respective time point are marked with asterisks (** $p < .01$, *** $p < .001$).

Supplementary Figure 2

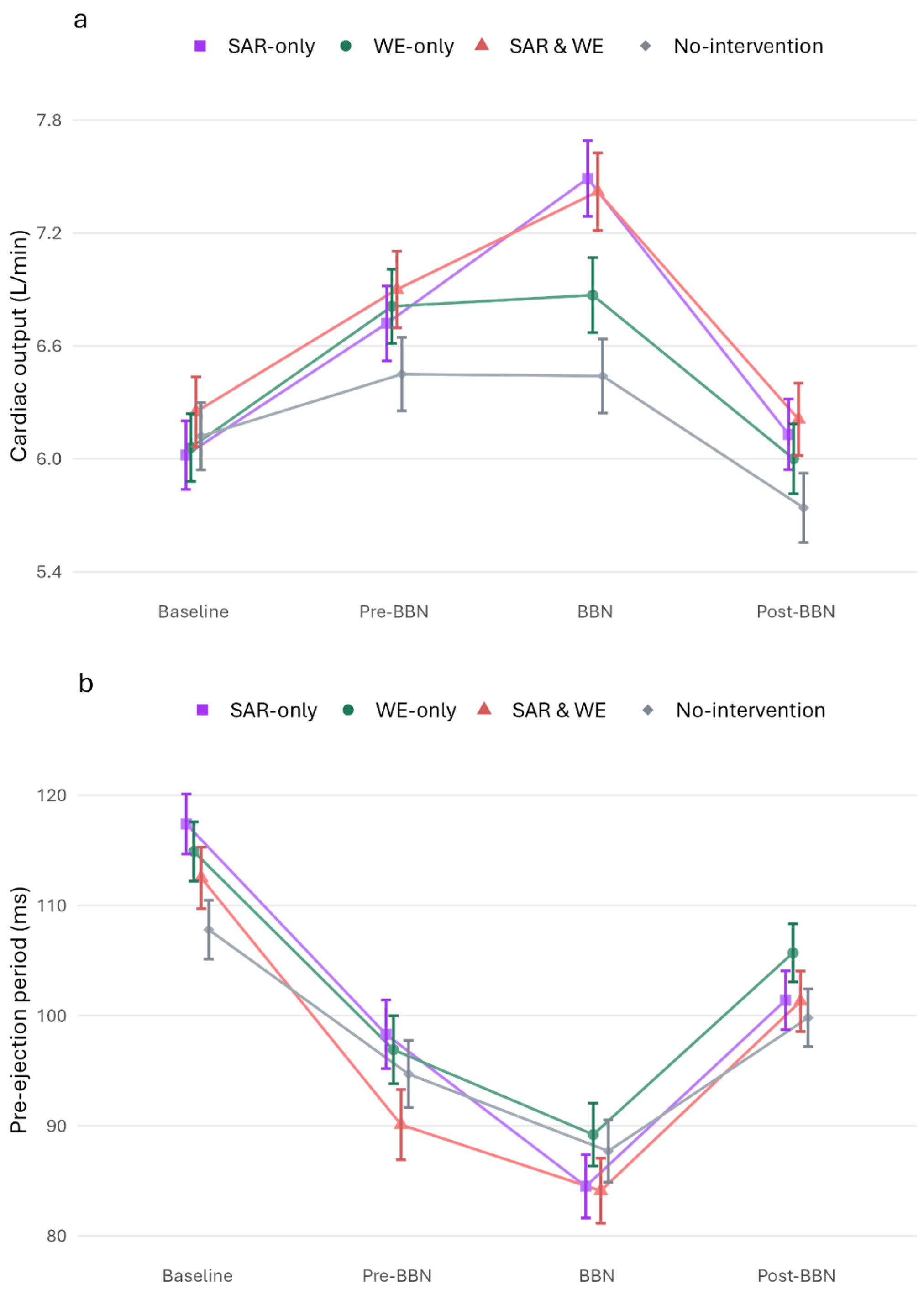

Line plots of Model 2 estimated marginal means of cardiac output (a) and pre-ejection period (b) for the SAR-only, WE-only, SAR & WE, and No-intervention groups. The error bar represents the standard error. For contrast effects, please see the Supplementary Tables S7 (cardiac output) and S12 (pre-ejection period).
